# Therapy by physician–pharmacist combination and economic returns for cancer pain management in China: a cost-effectiveness analysis

**DOI:** 10.3389/fphar.2023.1073939

**Published:** 2023-08-04

**Authors:** Xikui Lu, Lu Zhang, Hangxing Huang, Xiangping Wu, Zhenting Wang, Ling Huang, Jingyang Li, Huimin Yu, Hongyan Zhang, Jian Xiao

**Affiliations:** ^1^ Department of Pharmacy, Xiangya Hospital, Central South University, Changsha, China; ^2^ National Clinical Research Center for Geriatric Disorders, Xiangya Hospital, Central South University, Changsha, China; ^3^ College of Pharmacy, Dali University, Dali, China; ^4^ Department of Pharmacy, Fuwai Central China Cardiovascular Hospital, Zhengzhou, China

**Keywords:** cancer pain, physician-pharmacist, cost-effectiveness analysis, decision trees, economics

## Abstract

**Objective:** To examine whether joint management of cancer pain by physicians and pharmacists in clinics provides economic advantages from the perspective of the Chinese healthcare system.

**Methods:** From February 2018 to March 2020, 100 patients who visited the joint cancer pain clinic at the Xiangya Hospital of Central South University were included. These patients were randomly assigned to either the control or intervention groups. The control group received regular outpatient services from a physician, while the intervention group received regular outpatient services from a physician and medication education provided by a pharmacist. The study considered various direct costs, including drug expenses, physician-pharmacist outpatient services, adverse event management, consultations, examinations, and readmissions. The outcome indicators considered were the cancer pain control rate and the reduction in pain scores. Decision tree modeling, single-factor sensitivity analysis, and probabilistic sensitivity analysis were performed to evaluate the cost-effectiveness of joint physician-pharmacist outpatient services compared to physician-alone outpatient services.

**Results:** The intervention group showed a significantly higher cancer pain control rate than the control group (0.69 vs. 0.39, *p* = 0.03). In the decision tree model, the intervention group had a significantly lower pain score than the control group (0.23 vs. 0.14). The cost per person in the intervention group was $165.39, while it was $191.1 per person in the control group. The univariate sensitivity analysis showed that the cost of self-management for patients in the control group was identified as the primary sensitivity factor. Probabilistic sensitivity analysis indicated that the joint clinic group had a favorable incremental cost-effectiveness compared to the physician clinic group. In addition, the probabilistic sensitivity analysis demonstrated an absolute advantage in the incremental cost-effectiveness of the joint clinic group over the outpatient physician group.

**Conclusion:** The participation of pharmacists in joint cancer pain clinic services led to improved pain management for patients, demonstrating a clear advantage in terms of cost-effectiveness.

## 1 Introduction

Pain is closely associated with cancer and is a significant diagnostic factor (Copenhaver et al., 2021). The global burden of cancer is projected to reach 28.4 million cases by 2040 (Sung et al., 2021). According to a forecast study, it is estimated that China alone will have approximately 4.82 million new cancer cases and 3.21 million cancer-related deaths by 2022 (Xia et al., 2022). In the 2021 China National Statistical Yearbook, malignant tumors are identified as the third leading cause of mortality in urban and rural regions of the country, accounting for 25.4% and 23.11% of the total mortality rate, respectively (National Bureau of Statistics, 2022). Cancer and AIDS are recognized as the most burdensome diseases on a global scale (Can et al., 2019).

Based on a comprehensive literature review over the last 40 years, 64% of patients with advanced or metastatic cancer experience pain, rising to 59% among those undergoing anticancer treatment. Furthermore, one-third of patients experience pain after achieving a tumor cure (van den Beuken-van Everdingen et al., 2007). Cancer pain compromises patient comfort and profoundly impacts their daily activities, relationships with family and friends, and overall quality of life (Swarm et al., 2019). Emerging evidence suggests that early and effective palliative care, including comprehensive pain management, is closely associated with improved quality of life and survival outcomes for cancer patients (Swarm et al., 2019).

The management of cancer pain is a complex task due to various factors. These include the intricate evaluation of cancer pain medications, substantial differences in drug tolerance among individuals, and the necessity for precise dose titration (Feng et al., 2022). Furthermore, using combination drugs increases the risk of potentially serious adverse reactions, further complicating pain management in cancer patients. To address the challenges of cancer pain management, China has established specialized facilities such as cancer pain management wards, multidisciplinary cancer pain management clinics, and general pain clinics (Yu et al., 2017). As early as 2011, the National Health Commission established cancer pain demonstration wards collaborating with oncology, pain, pharmacy, and other departments to collectively manage cancer pain patients. However, one limitation of these management approaches is that healthcare professionals often prioritize patient diagnosis, adjustment of the medication regimen, and treatment outcomes, inadvertently neglecting to adequately educate patients about their understanding of cancer pain and the medications used for its treatment. This oversight frequently leads to under-treatment of cancer pain in patients (Su et al., 2021).

Clinical studies have highlighted the valuable contribution of clinical pharmacists to improving pain outcomes through enhanced communication, pain assessment, and patient education (Martinez et al., 2014; Adam et al., 2015). Recognizing the importance of their role, the National Health Commission in China initiated the training of clinical pharmacists in 2006 and established pain professional training centers. After a year of pain orientation training, pain pharmacists are equipped to conduct drug reviews, provide patient education and counseling, detect and manage adverse drug reactions (ADRs), recommend dose or treatment adjustments to physicians, and perform cancer pain assessments (Shrestha et al., 2023). Pharmacists play an active and significant role in facilitating the transition of care for patients with cancer pain. A study revealed that implementing a cancer pain management application by clinical pharmacists resulted in notable reductions in pain levels and readmission rates for outpatients experiencing cancer pain (Zhang et al., 2021). A meta-analysis conducted by Shrestha et al. (2022) further demonstrated that pharmacist involvement in cancer pain management, which includes medication review, patient education, monitoring and managing ADRs, providing pharmacologic recommendations (dose and drug therapy selections), and pain assessment, significantly reduced pain intensity, minimized adverse effects, and improved patient quality of life. However, it should be noted that the participation of pharmacists in cancer pain management can increase the cost of patient visit services, thus adding to the overall burden of the disease for patients.

The cost-effectiveness of pharmacists in cancer pain management within the Chinese healthcare system is uncertain. Therefore, this study aimed to assess the cost-effectiveness of integrating pharmacists into the team responsible for managing cancer pain.

## 2 Methods

### 2.1 Study design

Our team conducted a clinical trial in the early stages, and the study was registered at Chictr.org with the registration number ChiCTR1900023075 (Liu, 2019). The trial focused on ambulatory patients with cancer pain who received care in a tertiary hospital. Participants were randomly assigned to either a control or intervention group using an allocation ratio of 1:1 using a random number table.

The target population was individuals who met the following criteria: 1) 18 years or older, 2) diagnosed with malignant tumors confirmed by pathological or cytological methods, 3) experienced cancer-related pain that met the diagnostic criteria for cancer pain outlined in the National Comprehensive Cancer Network (NCCN) guidelines and was categorized as moderate to severe (Numeric Rating Scale [NRS] ≥ 4), 4) capable of reading and using WeChat (the largest social networking app in China) by the patient or family members, 5) possessed the normal verbal ability and performance status, and 6) provided a voluntary agreement to participate in the study and signed the informed consent form.

Perspective and interventions/comparator: only the medical costs incurred within the hospital were considered. The study investigated the economic viability of incorporating pharmacy services in the given context. The subjects were divided into two groups: the intervention group, which received interventions from physicians and pharmacists, and the control group, which received interventions solely from physicians.

Care of patients in the intervention group: each day, the pharmacist collected and recorded data in the 24-h pain diary, documenting patients’ pain levels and any related observations. Every 3 days, the pharmacist also completed adverse drug reaction (ADR) forms, noting any adverse effects experienced by the patients. Additionally, every 15 days, the pharmacist administered a Brief Pain Inventory (BPI) form to assess pain intensity and its impact on the patient’s daily life. Based on this comprehensive review, the pharmacist proposed appropriate pharmacological interventions to the attending physician if a change in medication, dosage, or treatment of an ADR was necessary. The physician was responsible for prescribing or adjusting the patients’ pain medications based on the pharmacist’s recommendations. In the cases where no changes were required, the pharmacist provided targeted education to address any knowledge deficits identified in the patients.

Care of the patients in the control group: the patients in the control group received conventional care. Before discharge, the pharmacist provided detailed medication education to ensure that patients understood their prescribed medications well. However, unlike the intervention group, the control group received no specific reminders or prompts to complete the forms mentioned earlier.

The time horizon was determined based on several factors. According to the NCCN guidelines, aiming to effectively control cancer pain within 24 h is recommended, with ideally completed opioid titration within 3 days. Considering patient adherence and cost measurement, the study duration was set at 4 weeks. Given the relatively short period of the study, we did not consider a discount rate.

Outcome evaluation: the primary outcomes were pain intensity and cancer-related pain control rate, while the secondary outcomes examined ADRs and readmission rates.

### 2.2 Control rate of cancer-related pain

The NRS score of a cancer patient <4 indicates that the patient has effectively controlled cancer pain. The calculation for the control of cancer pain rate is as follows: Control of cancer pain rate = (number of patients with NRS <4 during the observation period/total number of patients undergoing cancer pain treatment during the same period) * 100%.

### 2.3 Cost-effectiveness analysis

#### 2.3.1 Decision tree

**FIGURE 1:**
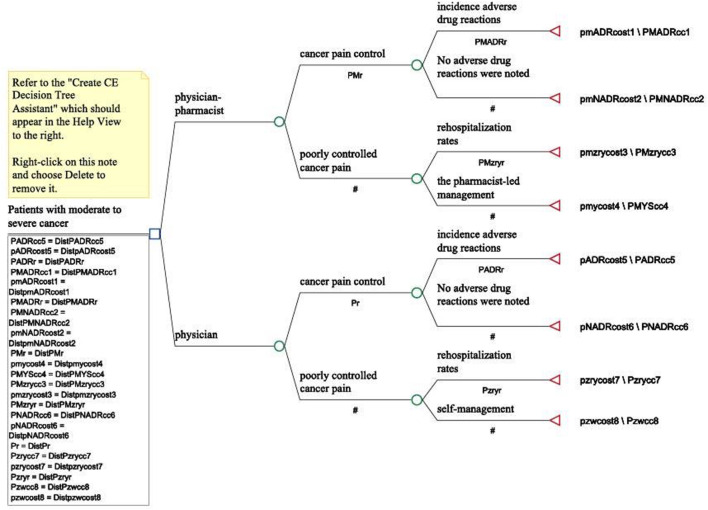
Decision-tree model for moderate to severe cancer pain.

Figure 1 shows the decision tree used in this study, illustrating the classification of subjects into two groups: physician-only intervention and physician-pharmacist joint intervention. Our model was designed to transition to one of the following scenarios: cancer-related pain control, occurrence of ADRs, readmission, pharmacist services, and patient self-management.

#### 2.3.2 Medical expenditure

Healthcare expenditure involved various elements, including expenses related to cancer pain treatment drugs, ADR treatment, laboratory tests, physician-pharmacist service fees, and pharmacist service fees. The cost of medications was derived from the 2018 Central South University Xiangya Hospital procurement system, accounting for the consultation and laboratory test fees. The expenses of pharmacist follow-up were calculated based on the average salary of pharmacists at the Xiangya Hospital of Central South University. The follow-up duration was determined using a median follow-up time of 10 min, costing $2.1 per session. Additionally, the pharmacist provided patient information and utilized the WeChat remote guidance service, which incurred charges equivalent to fees set by the Internet Clinic at the hospital, amounting to USD 1.479 per session. Details are shown in Tables 1, 2.

**TABLE 1:** The cost of drugs, tests, and consultation services.

Items	Specification	Unit price ($)
Drugs		
Oxycodone and acetaminophen Tablet	0.33 g × 10 pills	5.97
Tramadol Hydrochloride Sustained-Release Tablets	100 mg × 10 pills	5.33
Fentanyl Transdermal Patches	4.2 mg × 5 pills	51.8
Morphine Hydrochloride Tablets	5 mg × 20 pills	2.1
Oxycodone Hydrochloride Controlled-release Tablets	10 mg × 10 pills	11.59
Morphine Hydrochloride Sustained-release Tablets	30 mg × 10 pills	11.42
Morphine Hydrachloride Injection	10 μg	0.5
Codeine Phosphate Tablets	15 mg × 20 pills	1.4
Imrecoxib Tablets	0.1 g × 10 pills	6.93
Flurbiprofen Cataplasms	40 mg × 6 pills	8.14
Gabapentin Capsules	0.3 g × 10 pills	1.61
Mecobalamin Tablets	0.5 g × 20 pills	3.95
Lactulose Oral Solution	100 mL:66.7 g	4.82
Diclofenac Sodium Sustained Release Tablets	75 mg × 10 pills	2.9
Celecoxib Capsules	0.2 g × 6 pills	4.57
Inspection Items		
Routine Blood Test	time	2.24
Kidney Function	time	2.52
Hepatic Function	time	7.7
Service Fees		
Physician-pharmacist clinic service fees	time	16.52
Physician clinic service fees	time	10.92

**TABLE 2:** Cost comparison between the two study groups.

Items	Intervention group ($)	Control group ($)
Total costs	142.89	131.70
Opioid costs	66.89	66.89
Opioid + Adjunctive therapy costs	76.69	97.40
Costs of treatment in case of adverse reactions	144.13	172.97
Costs of treatment without adverse reactions	146.67	99.23
Readmission fees	152.36	267.70
Self-management costs	396.97	167.26

Note: Patient self-management cost refers to the cost of controlling cancer pain when there is no intervention of medical staff at home.

When prescribed drugs did not effectively control patients’ pain, they could either be readmitted to the hospital or contact the pharmacist for assistance. Through pharmacy services, the pharmacist focused on enhancing patient adherence and addressing any ADR using WeChat as a communication platform. The calculations were based on the hospital Internet outpatient charges to determine the costs involved. The readmission fees were obtained from the patient discharge invoice data.

#### 2.3.3 Cancer treatment effect

The effectiveness of the treatment was evaluated based on the reduction in the NRS score. An NRS score less than 4 at the final follow-up was considered effective, and treatment efficiency was estimated separately for each scenario. When comparing the two groups’ efficiency, the least-cost analysis method was utilized if the difference was statistically significant. If the difference was statistically significant, a cost-effectiveness analysis was performed. A decision-tree model was established to guide the study. A decrease in the NRS score indicated effective treatment and was assigned a value of 1, while an increase or no change in the NRS score indicated ineffective treatment and was assigned a value of 0. The treatment efficiency was then calculated for each scenario by dividing the number of effective cases by the total number of patients in the group.

#### 2.3.4 Cost-effectiveness analysis

When assessing multiple programs, a comparison was made between the costs and effects of two intervention programs. If one program demonstrated a higher effect and a lower cost than the other, it indicated a clear advantage in achieving a greater effect at a lower cost.

### 2.4 Sensitivity analyses

We conducted a one-factor sensitivity analysis to examine the impact of varying each state parameter within its designated range of values on the study outcomes. The medication cost was considered, with upper and lower limits representing the actual costs incurred by the patients. A range of probabilities was applied, with values set 20% higher or lower than the base value. The results of the single-factor sensitivity analysis were presented in the form of a cyclone diagram. We performed a probabilistic sensitivity analysis to further explore the impact of each state parameter on the study outcomes. This analysis involved randomizing each parameter according to its distribution pattern. The Monte Carlo simulation method was used, repeating the extraction simulation 10,000 times. The cost followed a Gamma distribution, while the effect and transfer probability followed a Beta distribution. The results of the probabilistic sensitivity analysis were presented in the form of cost-effectiveness acceptability curves and scatter plots on the cost-effectiveness plane, as detailed in Table 3.

**TABLE 3:** The key model parameter.

Items	Assign	Lower value	Upper value	Distribution	Source
Outputs-NRS reduced to effective (%)					
Adverse reactions occurred in the intervention group	0.37	0.296	0.444	Beta	Zhang et al. (2021)
No adverse effects occurred in the intervention group	0.12	0.096	0.144	Beta	Zhang et al. (2021)
Intervention group readmission	0.06	0.048	0.072	Beta	Zhang et al. (2021)
Intervention group pharmacist adjustment program services	0.08	0.064	0.096	Beta	Zhang et al. (2021)
Adverse reactions occurred in the control group	0.06	0.048	0.072	Beta	Zhang et al. (2021)
No adverse reactions in the control group	0.22	0.176	0.264	Beta	Zhang et al. (2021)
Control group readmission	0.12	0.096	0.144	Beta	Zhang et al. (2021)
Patients in the control group adjusted their own protocol	0.12	0.096	0.144	Beta	Zhang et al. (2021)
Probabilities (%)					
Cancer pain control rate in the intervention group	0.69	0.552	0.828	Beta	Zhang et al. (2021)
Control rate of cancer pain control in the control group	0.39	0.312	0.468	Beta	Zhang et al. (2021)
Intervention group readmission rate	0.76	0.608	0.912	Beta	Zhang et al. (2021)
Readmission rate in the control group	0.63	0.504	0.756	Beta	Zhang et al. (2021)
Incidence of adverse reactions in the intervention group	0.71	0.568	0.852	Beta	Zhang et al. (2021)
Incidence of adverse reactions in the control group	0.41	0.328	0.492	Beta	Zhang et al. (2021)
Costs ($)					
Costs of adverse reactions in the intervention group	144.13	126.04	312.49	Gamma	Medicine Procurement System, Medical Insurance
Costs of no adverse effects in the intervention group	146.67	128.09	178.84	Gamma	Medicine Procurement System, Medical Insurance
Intervention group readmission costs	152.36	108.54	190.77	Gamma	Medicine Procurement System, Medical Insurance
Intervention group pharmacist adjusts program costs	396.97	113.91	482.75	Gamma	Medicine Procurement System, Medical Insurance
Costs of adverse reactions in the control group	172.97	52.02	229.79	Gamma	Medicine Procurement System, Medical Insurance
No adverse reaction costs in the control group	99.23	79.70	140.90	Gamma	Medicine Procurement System, Medical Insurance
Costs of readmission for the control group	267.65	132.94	398.86	Gamma	Medicine Procurement System, Medical Insurance
Control group self-management costs	167.26	73.15	488.36	Gamma	Medicine Procurement System, Medical Insurance

## 3 Results

### 3.1 Outcome of cancer-related pain efficacy and adverse effects

In the intervention group, cancer-related pain control rates, incidence of ADRs, and adherence with the prescribed intervention were significantly higher compared to the control group, with statistically significant differences (Table 4). However, the two groups observed no statistically significant differences in readmission rates.

**TABLE 4:** Cancer-pain treatment effect and adverse drug reactions.

Variable	Intervention group (*n* = 51)	Control group (*n* = 49)	*p*-Value
Cancer pain control, n (%)	35 (68.63)	19 (38.78)	0.03 (χ^2^ = 8.965)
Rehospitalization rates, n (%)	39 (76.47)	31 (63.27)	0.15 (χ^2^ = 2.075)
Incidence adverse drug reactions, n (%)	36 (70.59)	20 (40.82)	0.03 (χ^2^ = 8.99)
Adherence, n (%)	21 (41.18)	16 (32.65)	0.377 (χ^2^ = 0.779)

### 3.2 Analysis results

#### 3.2.1 Basic analysis results

Cost-effectiveness analysis involves quantifying the monetary cost of an alternative concerning its associated benefits, which are expressed as clinical outcome indicators. After calculating the costs using the model, the intervention group had a cost of $165.39 and an effect of 22.62%. In contrast, the control group had a cost of $191.1 and an effect of 13.87%. This analysis shows that the intervention group had lower costs and achieved better outcomes, making it more cost-effective (Table 5).

**TABLE 5:** The results of the basic analysis.

Group	C($)	E(%)	C/E	ICER
Intervention group	165.39	22.62	7.31	-
Control group	191.10	13.87	13.78	-

Note: Intervention group is dominance.

#### 3.2.2 Single-factor sensitivity analysis results

**FIGURE 2:**
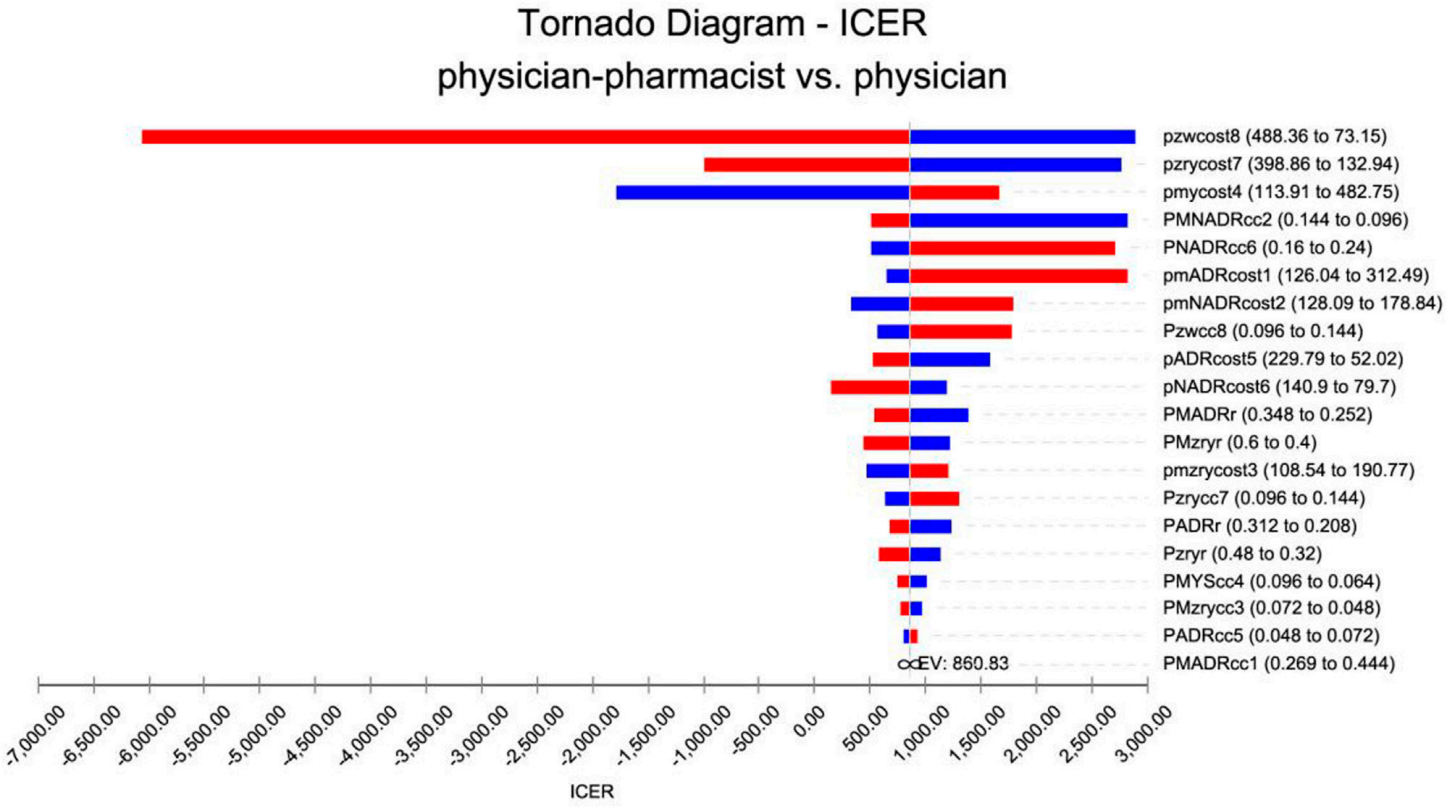
Tornado diagram for one-way sensitivity analysis. pmADRcost1: The cost of adverse reactions in the intervention group. pmNADRcost2: The cost of no adverse effects in the intervention group. pmzrycost3: The readmission costs in the intervention group. pmycost4: The cost of pharmacist services in the intervention group. pADRcost5: The cost of adverse reactions in the control group. pNADRcost6: The cost of no adverse effects in the control group. pzrycost7: The cost of readmission in the control group. pzwcost8: The cost of pharmacist services in the control group. PMADRcc1: The treatment efficiency of adverse reactions in the intervention group. PMNADRcc2: The treatment efficiency of no adverse reactions in the intervention group. PMzrycc3: The readmission treatment efficiency in the intervention group. PMYScc4: The pharmacist-adjusted regimen service treatment efficiency in the intervention group. PADRcc5: The treatment efficiency of adverse reactions in the control group. PNADRcc6: The treatment efficiency of no adverse reactions in the control group. Pzrycc7: The readmission treatment efficiency in the control group. Pzwcc8: The pharmacist-adjusted regimen service treatment efficiency in the control group. PMzryr: The readmission rate in the intervention group. Pzryr: The rate of readmission in the control group. PMADRr: The incidences of adverse reactions in the intervention group. PADRr: The incidences of adverse reactions in the control group.

A univariate sensitivity analysis was conducted on medical costs, treatment efficiency, adverse effect control, and readmission rates. The analysis revealed that the most influential factors affecting the outcome were the costs associated with readmission from control patients and the costs related to self-management by control patients. These findings are visually presented in a cyclone diagram depicted in Figure 2.

#### 3.2.3 Results of the probabilistic sensitivity analysis

**FIGURE 3:**
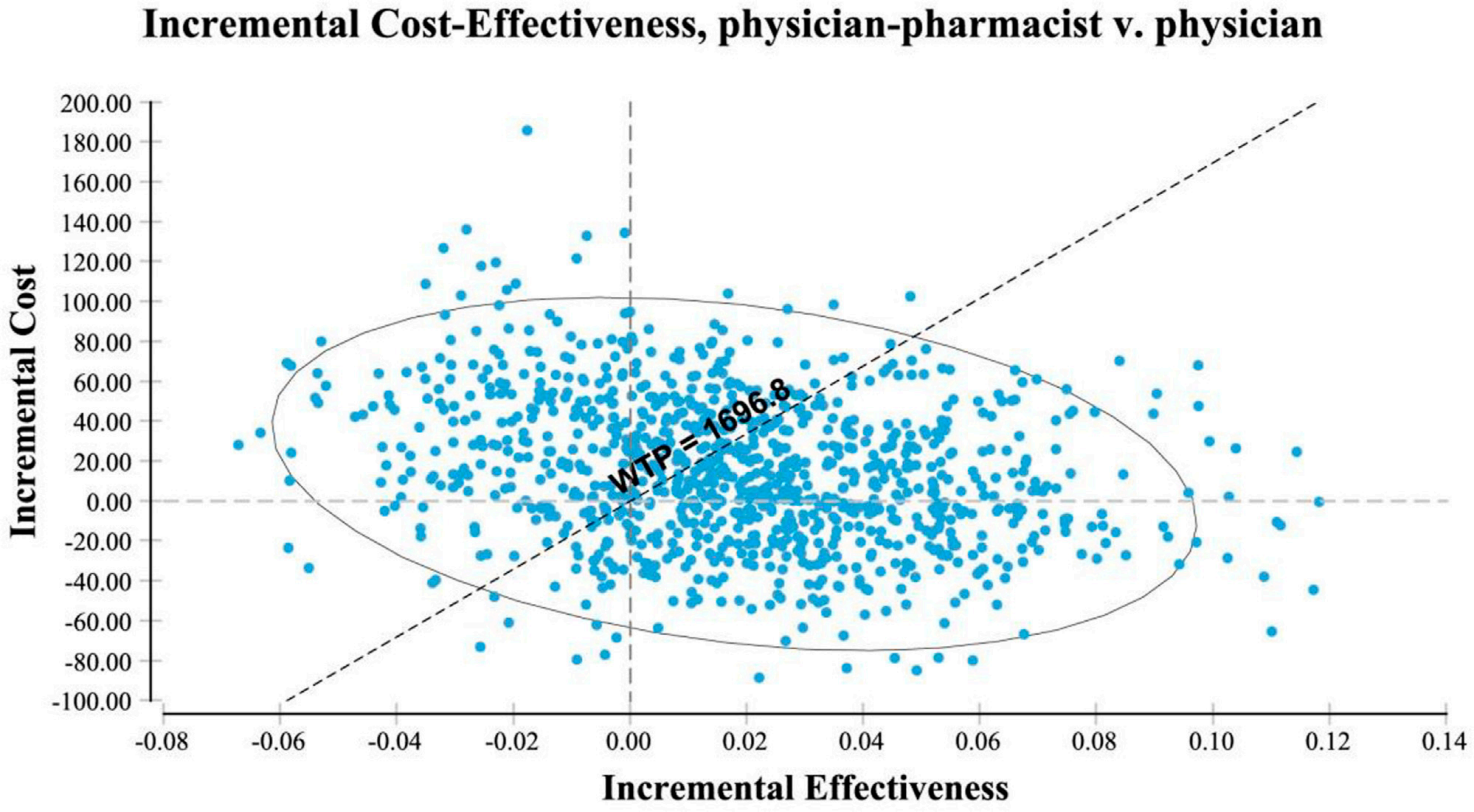
Plane scatter plot of incremental cost-effectiveness.

A planar scatter plot of incremental cost-effectiveness was generated by performing 10,000 repetitions of the simulation using Monte Carlo simulation. Figure 3 illustrates the scatter plot, where the origin represents the outpatient physician group. The scattered representative medicines combined with the outpatient group are plotted with their corresponding incremental cost-effectiveness ratios (ICER). It can be observed that the majority of ICERs in the figure lie in quadrant 4, indicating that the joint clinic approach is not only cost-effective but also provides an absolute economic advantage, resulting in cost savings.

**FIGURE 4:**
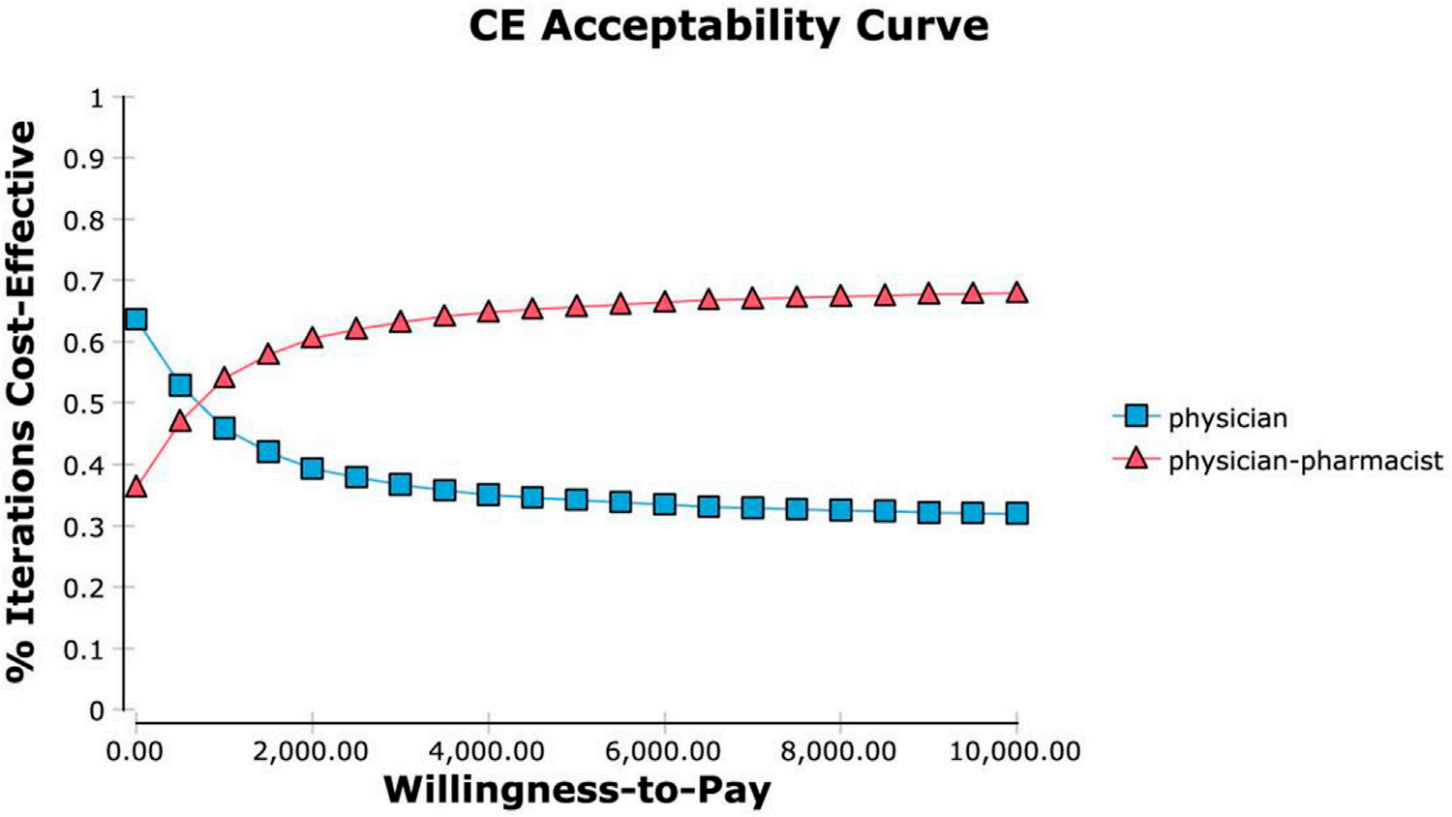
Cost-effectiveness acceptability curve.

The economic analysis of the physician-pharmacist group was performed by plotting a cost-utility acceptability curve. In Figure 4, the horizontal axis represents the willingness-to-pay (WTP) value, while the vertical axis represents the probability that the intervention is more cost-effective. Based on the results, when the WTP threshold of a patient was set at $300, there was an 86% probability that the intervention group was more economical than the control group. In contrast, when the WTP threshold exceeded $1500, the intervention group exhibited a 95% probability of being more cost-effective than the control group.

#### 3.2.4 Different management methods to save medical expenses for cancer pain

According to the latest data from CANCER TODAY (World Health Organization, 2022), the total number of cancer cases in China in 2020 was 4,568,754. Among these cases, 64% reported experiencing cancer pain. Based on our current findings, the intervention group achieved equivalent treatment outcomes while saving $6.33 per person per month. Extrapolating this over 3 years, cancer patients experiencing pain could save a total of $104 million.

## 4 Discussion

Morbidity and mortality rates in patients with advanced cancer remain persistently high. However, the availability of a broader range of treatment options for advanced cancer has led to improved treatment efficacy. In particular, the 5-year survival rate among patients with advanced cancer has increased significantly, resulting in a notable increase in patients living with tumors for extended periods. Despite these advances, advanced cancer patients frequently experience pain requiring opioid analgesics (Swarm et al., 2019). Currently, most cancer pain patients are being managed outpatient with opioids, but there are challenges to providing effective pain management for this patient population. Sun et al. (2017) reported significant differences in mean pain scores using an intelligent pain management system designed for these patients. Furthermore, a randomized controlled trial (Zhang et al., 2021) demonstrated that joint physician-pharmacist clinics, supported by the WeChat platform, could effectively manage cancer patients and optimize pain management outcomes.

Although comprehensive and multifaceted education has shown the potential to improve patient outcomes in cancer pain treatment, it simultaneously increases the financial burden on patients. In the management of opioids, pharmacists play a crucial role, including tasks such as pain assessment, suggesting adjustments to treatment protocols, identifying medication-related issues, guiding medication use, patient follow-up, and monitoring ADRs. Assessing the economic viability of involving pharmacists in cancer pain management is essential to determine its cost-effectiveness. This study analyzed the cost-effectiveness of joint outpatient physician-pharmacist management compared to outpatient physician management of cancer pain from the perspective of the Chinese healthcare system.

In the base analysis, the physician-pharmacist group demonstrated enhanced patient adherence and improved cancer pain control, even with the additional cost of pharmacist services. Decision-tree model analysis overwhelmingly supported the physician-pharmacist group as the superior option for managing cancer pain. During the univariate sensitivity analysis, the costs associated with the readmission of control group patients to the hospital emerged as the most influential factor, followed by the costs incurred by patients in self-management.

A previous study (Lovell et al., 2014) demonstrated the effectiveness of patient education in alleviating cancer pain distress. However, in China’s general outpatient clinics, the visit duration is among the shortest globally, typically lasting around 7 min (sometimes as fast as 2–3 min) (Or et al., 2018). This limited time frame restricts physicians’ ability to adequately educate patients and communicate effectively with them. Consequently, patients may have insufficient knowledge about their disease and medications, increasing self-management costs. To address this problem, pain pharmacists play a crucial role as the primary providers of pharmacy services. They can conduct pain assessments, design protocols for pain medication administration, provide medication education, and work toward improving patient control of cancer pain, medication adherence, reducing adverse reactions, and minimizing patient readmission rates. Informing patients about cancer pain and appropriate medication use can reduce readmission costs. In this study, WeChat was used for patient management. Pharmacists maintained regular communication by collecting 24-h pain diaries, ADR forms every 3 days, and BPI form every 15 days through WeChat. Patients also had the opportunity to communicate with medical staff through WeChat if they had any treatment-related questions. This approach was designed to enhance patient engagement and support effective pain management.

In this study, the incidence of ADRs was higher in the intervention group compared to the control group. This difference could be attributed to the intervention group’s proactive approach, which used WeChat to report ADRs every 3 days immediately. This regular reporting helped mitigate bias caused by forgetfulness. In contrast, the control group patients were only seen once every 2 weeks, and they often forgot to report ADRs in the absence of reminders, resulting in potential data loss. Patient awareness of ADRs is generally low. In the intervention group, pharmacists actively educated patients about medication use, making them aware of possible ADRs that might occur during treatment. For example, if a patient in the intervention group experienced dizziness, they were encouraged to complete the ADR registry. Patients in the control group often attributed such symptoms to the disease and did not inform physicians during outpatient follow-up visits. This study relied primarily on a randomized controlled trial conducted by the subject group to ensure that the data source was first-hand and reliable. The treatment effectiveness rate at each decision point served as an effect parameter, while the medical expenses incurred by the patients were considered costs. A comprehensive evaluation of the interventions’ cost-effectiveness was performed by calculating the ICER.

This study has some limitations. First, the sample size was relatively small, which could limit the generalizability of the findings and the statistical power of the analysis. Second, the data source was derived from a single center, specifically a large tertiary care hospital in China, which may not fully represent the experiences and practices of hospitals at different levels and in other countries. Third, the follow-up duration was relatively short, as cancer pain studies have reported mean survival times of 318 days (Reyes et al., 2019). Furthermore, because patients in the control group were followed once every 2 weeks, there may be a recall bias, leading to a lower reported incidence of ADR. Last, the lack of clinical studies on managing cancer pain in outpatient joint clinics globally limits the availability of upper and lower clinical treatment effect data thresholds. To address this, we incorporated a variety of treatment effects by using both 20% above and below the base value to account for the uncertainty in clinical outcomes.

## 5 Conclusion

Managing cancer pain patients through physician-pharmacist joint clinics is more effective and cost-effective than general physician office visits. This finding highlights the potential advantages and benefits of integrating pharmacists into the care team for managing cancer pain. The physician-pharmacist collaboration can contribute to improved patient outcomes and better utilization of healthcare resources, making it a favorable option for cost-effectiveness.

## Data Availability

The original contributions presented in the study are included in the article/Supplementary Material, further inquiries can be directed to the corresponding author.
