# Corrigendum: Therapy by physician–pharmacist combination and economic returns for cancer pain management in China: a cost-effectiveness analysis

**DOI:** 10.3389/fphar.2023.1327256

**Published:** 2023-11-17

**Authors:** Xikui Lu, Lu Zhang, Hangxing Huang, Xiangping Wu, Zhenting Wang, Ling Huang, Jingyang Li, Huimin Yu, Hongyan Zhang, Jian Xiao

**Affiliations:** ^1^ Department of Pharmacy, Xiangya Hospital, Central South University, Changsha, China; ^2^ National Clinical Research Center for Geriatric Disorders, Xiangya Hospital, Central South University, Changsha, China; ^3^ College of Pharmacy, Dali University, Dali, China; ^4^ Department of Pharmacy, Fuwai Central China Cardiovascular Hospital, Zhengzhou, China

**Keywords:** cancer pain, physician-pharmacist, cost-effectiveness analysis, decision trees, economics

In the published article, there is a missing **Funding** statement. The correct **Funding** statement appears below.

“The author(s) declare financial support was received for the research, authorship, and/or publication of this article. The study was supported by Hunan Provincial Natural Science Foundation of 2021 (2021JJ31043); Changsha Natural Science Foundation of 2020 (kq2007039); 2020 National Geriatric Disease Clinical Medicine Research Center Appropriate Technology Promotion Project (XYYYJSTG-15); Parallel and Distributed Processing for the Stable Support Project of the National Defense Science and Technology Key Laboratory (WDZC20205500121); Key Research Project of Ningxia Hui Autonomous Region in 2021 (Major Project) (2021BEG01001); Degree & Postgraduate Education Reform Project of Central South University (2022JGB067)”.

The authors apologize for this error and state that this does not change the scientific conclusions of the article in any way. The original article has been updated.

